# Chest X-Ray evaluation using GPT for tube thoracostomy or conservative care in non-tension spontaneous pneumothorax

**DOI:** 10.1186/s13049-026-01616-2

**Published:** 2026-04-17

**Authors:** Ertuğ Günsoy, Ahmet Aykut, Cem Yıldırım, Mehmet Veysel Öncül, Saim Türkoğlu

**Affiliations:** 1 Emergency Department, Van Education and Research Hospital, Van, Turkey; 2 Faculty of Medicine, Department of Radiology, Yuzuncu Yıl University, Van, Turkey

**Keywords:** Spontaneous pneumothorax, Chest radiography, Tube thoracostomy, ChatGPT, Large language models

## Abstract

**Background/Objective:**

Spontaneous pneumothorax (SP) commonly presents to the emergency department (ED), and clinicians must rapidly decide between conservative care and tube thoracostomy. Most artificial intelligence tools focus on pneumothorax detection rather than quantitative size estimation and actionable management recommendations. We aimed to evaluate whether a vision-enabled generative pre-trained transformer (GPT) model could estimate apical SP depth on chest radiographs (CXRs) and predict initial ED management.

**Methods:**

We conducted a single-center retrospective observational study of adult patients (≥ 18 years) with confirmed SP on pre-intervention posteroanterior CXR (January 1, 2023–December 31, 2025). GPT received only the CXR plus age and sex and returned laterality, estimated apical depth (Depth_cm), and a binary management recommendation (tube thoracostomy vs conservative). The reference outcome was tube thoracostomy within 24 h. We calculated diagnostic performance metrics and area under the receiver operating characteristic curve (AUC). We assessed agreement between GPT depth estimates and blinded radiologist measurements using intraclass correlation coefficient (ICC), Bland–Altman analysis, and mean absolute error (MAE).

**Results:**

Among 101 patients, mean age was 33.1 ± 14.6 years and 89 (88.1%) were male; 53 (52.5%) underwent tube thoracostomy within 24 h. GPT showed sensitivity 86.8% (95% CI, 75.2%–93.5%), specificity 93.8% (95% CI, 83.2%–97.9%), and accuracy 90.1% (95% CI, 82.7%–94.5%), with Cohen’s κ = 0.80 and AUC 0.90 (95% CI, 0.84–0.96). Depth agreement was strong (ICC, 0.893), with MAE 0.69 cm and mean bias −0.51 cm (95% limits of agreement, − 2.30 to 1.27 cm).

**Conclusions:**

In confirmed SP, a vision-enabled GPT model produced apical depth estimates that closely agreed with radiologist measurements and generated management recommendations that substantially matched real-world ED decisions, supporting its potential role as adjunct imaging decision support.

**Supplementary Information:**

The online version contains supplementary material available at 10.1186/s13049-026-01616-2.

## Introduction

Spontaneous pneumothorax (SP) remains a frequent reason for emergency department (ED) presentation and hospital admission, affecting both younger patients without known lung disease and older, comorbid patients with secondary spontaneous pneumothorax (SSP). SP causes acute pleuritic pain and dyspnea, drives repeat healthcare utilization because recurrence is common, and can be associated with substantial morbidity and mortality in SSP. Population-level analyses also show meaningful recurrence risk after an index event, particularly among younger patients and those who undergo tube thoracostomy, reinforcing the clinical importance of accurate early assessment and risk-stratified management [1, 2].

Current guidance supports individualized management based on symptoms, physiologic stability, and imaging, yet real-world decision-making remains heterogeneous across specialties and institutions. The 2023 British Thoracic Society (BTS) pleural disease guideline reflects evolving practice toward less invasive strategies in selected patients, but clinicians still rely heavily on chest radiographs (CXRs) and size estimation when choosing observation versus intervention [1, 3]. In parallel, artificial intelligence (AI) tools now improve pneumothorax detection and reader performance on CXR, including ED-facing multi-reader studies and workflow evaluations [4, 5]. However, most prior AI work focuses on binary detection or triage rather than quantitative measurement and explicit management recommendation, which are the steps clinicians must operationalize at the bedside. Emerging multimodal large language models (LLMs) can interpret CXRs and generate radiology-style outputs, but evidence remains limited regarding their reliability for clinically actionable tasks in acute care contexts [6, 7].

The aim of this study was to evaluate a vision-enabled generative pre-trained transformer (GPT) model for CXR-based decision support in confirmed spontaneous pneumothorax. Specifically, we compared GPT-derived apical pneumothorax depth estimates with radiologist measurements and assessed GPT’s performance for predicting the initial ED management strategy (tube thoracostomy within 24 h vs conservative management) using the clinical decision as the reference standard. We hypothesized that GPT would demonstrate strong agreement with radiologist measurements and clinically meaningful discrimination for initial management decisions.

## Methods

### Study design and setting

**Fig. 1:**
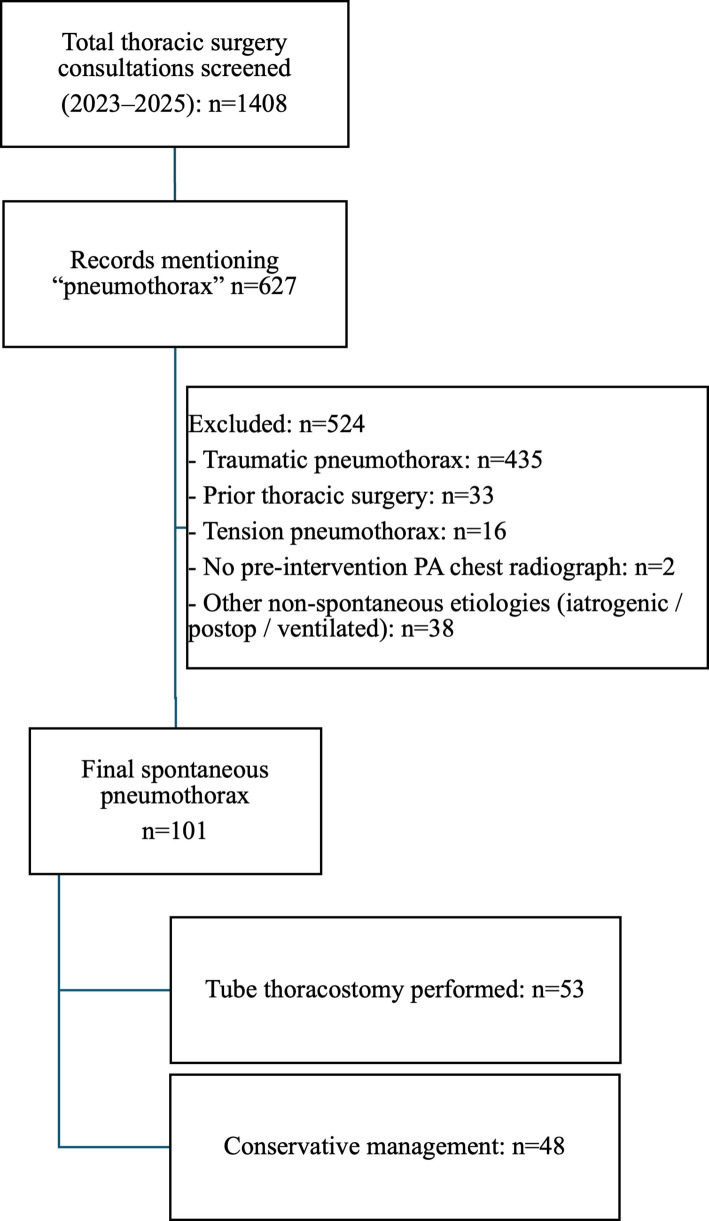
Flow diagram illustrating patient selection, exclusion criteria, and derivation of the final study cohort

This single-center retrospective observational study evaluated the performance of a vision-enabled large language model (GPT) in predicting initial clinical management decisions for patients with spontaneous pneumothorax. All thoracic surgery consultations requested from the adult emergency department between 1 January 2023 and 31 December 2025 were identified through the hospital information system. During the study period, 1,408 consultations were screened. After eligibility assessment and application of predefined exclusion criteria, all eligible adult patients with confirmed spontaneous pneumothorax during the study period were included in the final cohort (Fig. 1).

The study was conducted in accordance with the Declaration of Helsinki and reported in line with the Strengthening the Reporting of Observational Studies in Epidemiology (STROBE) recommendations. The institutional ethics committee approved the study protocol (Decision No.: GOKAEK-2025–09–16, Date: 07.11.2025), and the requirement for informed consent was waived owing to the retrospective design and full anonymization of all patient data.

### Eligibility criteria

All thoracic surgery consultations in adult patients (≥ 18 years) that were requested for suspected pneumothorax in the emergency department were retrospectively reviewed. Patients were included if spontaneous pneumothorax was confirmed on a posteroanterior (PA) chest radiograph obtained in the emergency department prior to any pleural intervention and if relevant clinical and imaging data were available in the electronic medical record.

Consultations were excluded if pneumothorax was classified as traumatic, tension, iatrogenic, or postoperative; if the patient had a history of prior thoracic surgery; if no pre-intervention PA chest radiograph was available; or if the consultation was ultimately judged not to represent true spontaneous pneumothorax. Tension pneumothorax was defined retrospectively based on contemporaneous clinical documentation indicating hemodynamic compromise in conjunction with radiographic findings. The number of cases excluded for each category and the derivation of the final cohort are summarized in Fig. 1.

### Data collection and variables

Clinical data, consultation records, and imaging studies were retrieved retrospectively from the hospital information system and the picture archiving and communication system (PACS). For each eligible patient, demographic variables (age and sex) and presenting clinical information were extracted for descriptive purposes. The initial PA chest radiograph obtained in the emergency department before any pleural intervention was used for all GPT evaluations.

The primary clinical outcome was whether tube thoracostomy was performed within the first 24 h following the index emergency department assessment. This measure reflects real-world clinical decision-making for initial management rather than a purely physiological gold standard for pneumothorax severity. Outcome status was determined using timestamped electronic orders, procedure notes, and operative reports and served as the reference standard for all diagnostic performance analyses. Management without tube thoracostomy (observation or conservative treatment) within the same 24-h window defined the non-intervention outcome. The 24-h time frame was selected to capture the initial definitive management decision following emergency department evaluation.

### Human reference standard for pneumothorax depth

To obtain a human reference for pneumothorax depth, a radiologist with experience in thoracic imaging, who was blinded to all GPT outputs and to the 24-h management outcome, independently reviewed each chest radiograph in PACS. The radiologist measured the apical interpleural distance in centimeters using digital calipers. This distance was defined as the perpendicular separation between the visceral pleural line and the chest wall at the lung apex on the upright PA chest radiograph and was used as a pragmatic human reference standard for pneumothorax depth estimation, acknowledging that CXR-based measurements are subject to interobserver variability. To contextualize expected human measurement variability, a second blinded radiologist independently re-measured apical depth in a random subset of 30 radiographs; inter-radiologist agreement was excellent (ICC 0.96; 95% CI 0.92–0.98).

All radiographs were exported from PACS in a uniform, lossless format with fixed resolution and windowing. No cropping, annotation, or additional image processing was performed prior to GPT evaluation.

### Model input standardization and blinding

The large language and vision model evaluated in this study was GPT-5.2 Instant (OpenAI), a vision-enabled large language model accessed via the ChatGPT platform in December 2025. To ensure reproducibility, a single fixed prompt template was developed a priori and applied identically to all cases; the full wording of the prompt is provided in Supplementary Material 1. The prompt template was finalized before data analysis and was not modified during GPT evaluation.

For each patient, GPT received only the PA chest radiograph obtained before any pleural intervention together with the patient’s age and sex. All radiographs were exported from PACS as lossless PNG files with fixed resolution and consistent windowing, and no preprocessing, compression, cropping, annotation, or additional image manipulation was performed prior to GPT evaluation. No symptoms, vital signs, laboratory data, or outcome information were supplied to GPT.

GPT was instructed to respond in a strictly structured three-line format:

1. pneumothorax laterality (right/left),
2. estimated apical pneumothorax depth in centimeters (Depth_cm), and.
3. a binary management recommendation indicating whether tube thoracostomy was required or conservative management was appropriate.

All evaluated cases represented confirmed spontaneous pneumothorax; therefore, GPT was not tasked with determining pneumothorax presence. Each case was evaluated in a separate session to prevent information leakage between cases. GPT had no access to information regarding subsequent clinical management or whether tube thoracostomy had actually been performed. All GPT responses were recorded verbatim without any manual editing or post-processing.

### Sample size considerations

For sample size estimation, a clinically relevant area under the receiver operating characteristic curve of approximately 0.80 was assumed for predicting the 24-h need for tube thoracostomy. Assuming a tube thoracostomy rate of approximately 50%, a minimum sample size of 90 patients was estimated to provide more than 80% power to detect an AUC around 0.80 at a two-sided alpha level of 0.05. The final cohort of 101 patients exceeded this prespecified minimum.

### Statistical analysis

All statistical analyses were performed using standard methods for the evaluation of binary classification and clinical decision prediction performance. The 24-h initial management strategy (tube thoracostomy versus conservative management) served as the reference standard. Sensitivity, specificity, positive and negative predictive values, overall accuracy, and the area under the receiver operating characteristic curve with 95% confidence intervals were calculated for GPT’s binary management recommendation. Agreement between GPT recommendations and the reference outcome was assessed using Cohen’s kappa coefficient.

For pneumothorax depth estimation, agreement between GPT-estimated apical pneumothorax depth (Depth_cm) and radiologist measurements was evaluated using a two-way mixed-effects intraclass correlation coefficient, Bland–Altman analysis, and mean absolute error. Continuous variables were summarized as mean ± standard deviation or median with interquartile range, as appropriate, while categorical variables were reported as counts and percentages. A two-sided p value < 0.05 was considered statistically significant. Statistical analyses were conducted using IBM SPSS Statistics (version 29.0; IBM Corp., Armonk, NY), Jamovi (version 2.5.5), and Python (SciPy and scikit-learn libraries).

## Results

A total of 101 adult patients with confirmed spontaneous pneumothorax were included in the final analysis (Fig. 1). The mean age of the study population was 33.1 ± 14.6 years, and 89 patients (88.1%) were male. Pneumothorax was located on the right side in 55 patients (54.5%) and on the left side in 46 patients (45.5%). Based on the reference clinical outcome, 53 patients (52.5%) underwent tube thoracostomy within the first 24 h following emergency department evaluation, whereas 48 patients (47.5%) were initially managed conservatively. Baseline demographic and radiographic characteristics of the study population, stratified by initial management strategy, are summarized in Table 1.

**Table 1:** Baseline demographic and radiographic characteristics of the study population stratified by initial management strategy

	All patients (*n* = 101)	Tube thoracostomy (*n* = 53)	Conservative management (*n* = 48)
Age, years (mean ± SD)	33.1 ± 14.6	35.6 ± 15.5	30.3 ± 13.1
Male sex, n (%)	89 (88.1%)	45 (84.9%)	44 (91.7%)
Right-sided pneumothorax, n (%)	55 (54.5%)	29 (54.7%)	26 (54.2%)
Radiologist-measured apical pneumothorax depth, cm (median [IQR])	3.00 [1.50–5.00]	5.00 [4.00–6.50]	1.50 [1.00–2.00]
GPT-estimated apical pneumothorax depth, cm (median [IQR])	2.00 [1.00–5.00]	5.00 [3.50–6.00]	1.00 [1.00–2.00]

Table 2 presents the confusion matrix and performance metrics for GPT’s prediction of the initial management strategy within the first 24 h, using the clinical management decision as the reference standard. GPT achieved a sensitivity of 86.8% (95% CI, 75.2%–93.5%) and a specificity of 93.8% (95% CI, 83.2%–97.9%), with an overall accuracy of 90.1% (95% CI, 82.7%–94.5%). The positive predictive value was 93.9% (95% CI, 83.5%–97.9%), and the negative predictive value was 86.5% (95% CI, 74.7%–93.3%). Agreement between GPT’s management recommendation and the reference outcome was high (Cohen’s κ = 0.80). GPT correctly identified pneumothorax laterality in all cases, consistent with the fact that all images represented confirmed spontaneous pneumothorax.

**Table 2:** Confusion matrix for GPT prediction of initial management strategy (tube thoracostomy versus conservative management) within 24 h

*n* = 101	Tube Thoracostomy (n)	Conservative Management (n)
GPT: Tube Thoracostomy	46 (True Positive)	3 (False Positive)
GPT: Conservative Management	7 (False Negative)	45 (True Negative)

Error pattern analysis showed that GPT recommended tube thoracostomy in three patients who were initially managed conservatively within the first 24 h (false positives). These cases generally represented borderline radiographic severity based on apical depth, and two of the three patients subsequently underwent tube thoracostomy after 24 h, suggesting evolving clinical or radiographic course. Initial conservative management may also have been influenced by non-radiographic factors (e.g., symptoms, patient preference, or local practice), which were not provided to the image-only model. In these false-positive cases, the median radiologist-measured apical pneumothorax depth was 5.0 cm (IQR, 4.0–5.5), while the median GPT-estimated depth was 4.0 cm (IQR, 4.0–5.0). In contrast, GPT recommended conservative management in seven patients who underwent tube thoracostomy within the first 24 h (false negatives). In the false-negative cases, the median radiologist-measured apical pneumothorax depth was 4.0 cm (IQR, 2.75–4.0), whereas the median GPT-estimated depth was 2.0 cm (IQR, 1.5–2.0).

**Fig. 2:**
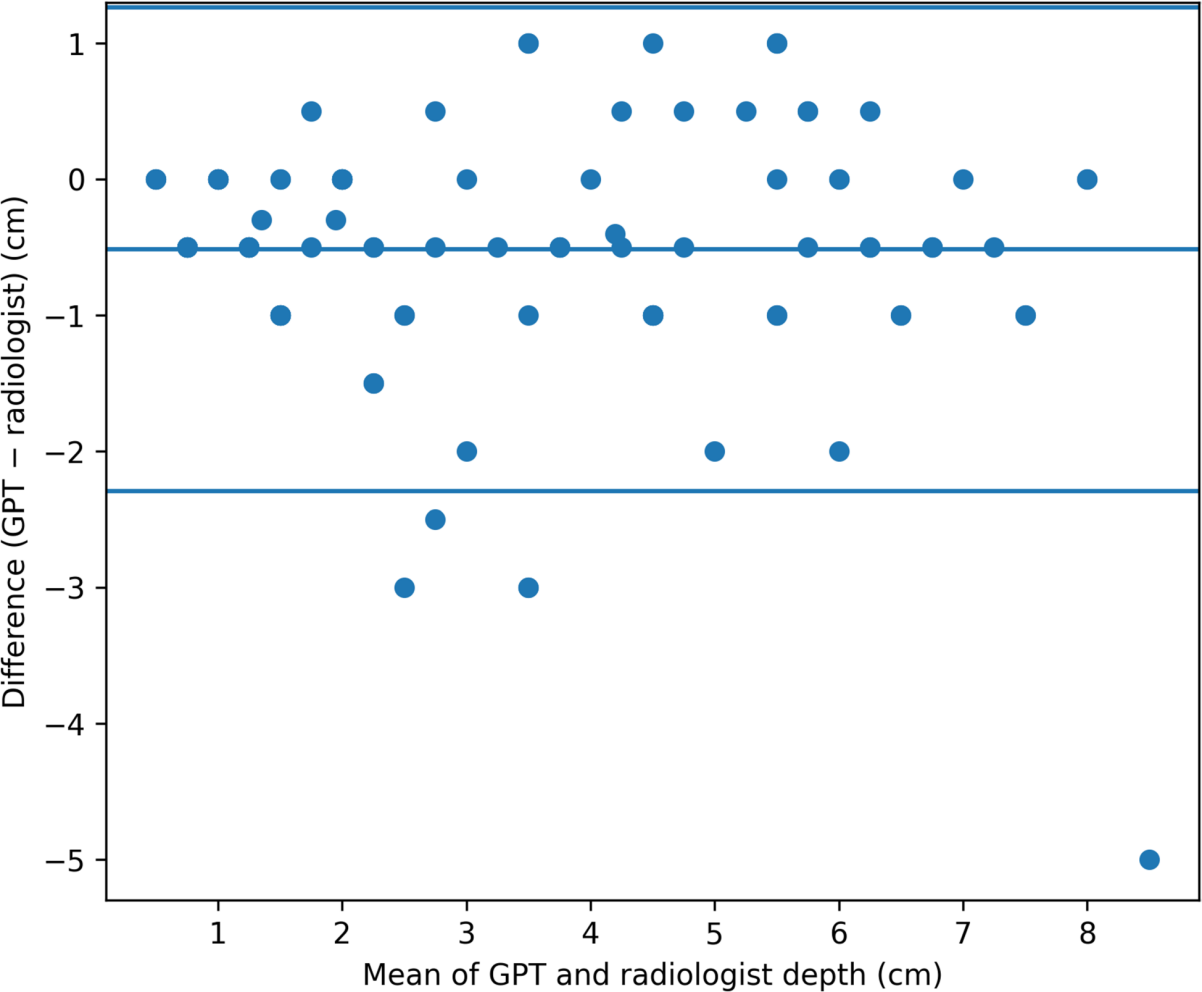
Bland–Altman plot for agreement between GPT-estimated and radiologist-measured apical pneumothorax depth. Bland–Altman plot demonstrating agreement between the GPT-estimated apical pneumothorax depth and radiologist measurements. The solid horizontal line represents the mean difference (bias), and the dashed lines indicate the 95% limits of agreement

Agreement between the GPT-estimated apical pneumothorax depth and radiologist measurements is illustrated in Fig. 2. GPT demonstrated strong agreement with the human reference standard, with a two-way mixed-effects intraclass correlation coefficient (ICC) of 0.893. The mean absolute error between GPT estimates and radiologist measurements was 0.69 cm. Bland–Altman analysis showed a mean bias of −0.51 cm, with 95% limits of agreement ranging from −2.30 to 1.27 cm.

**Fig. 3:**
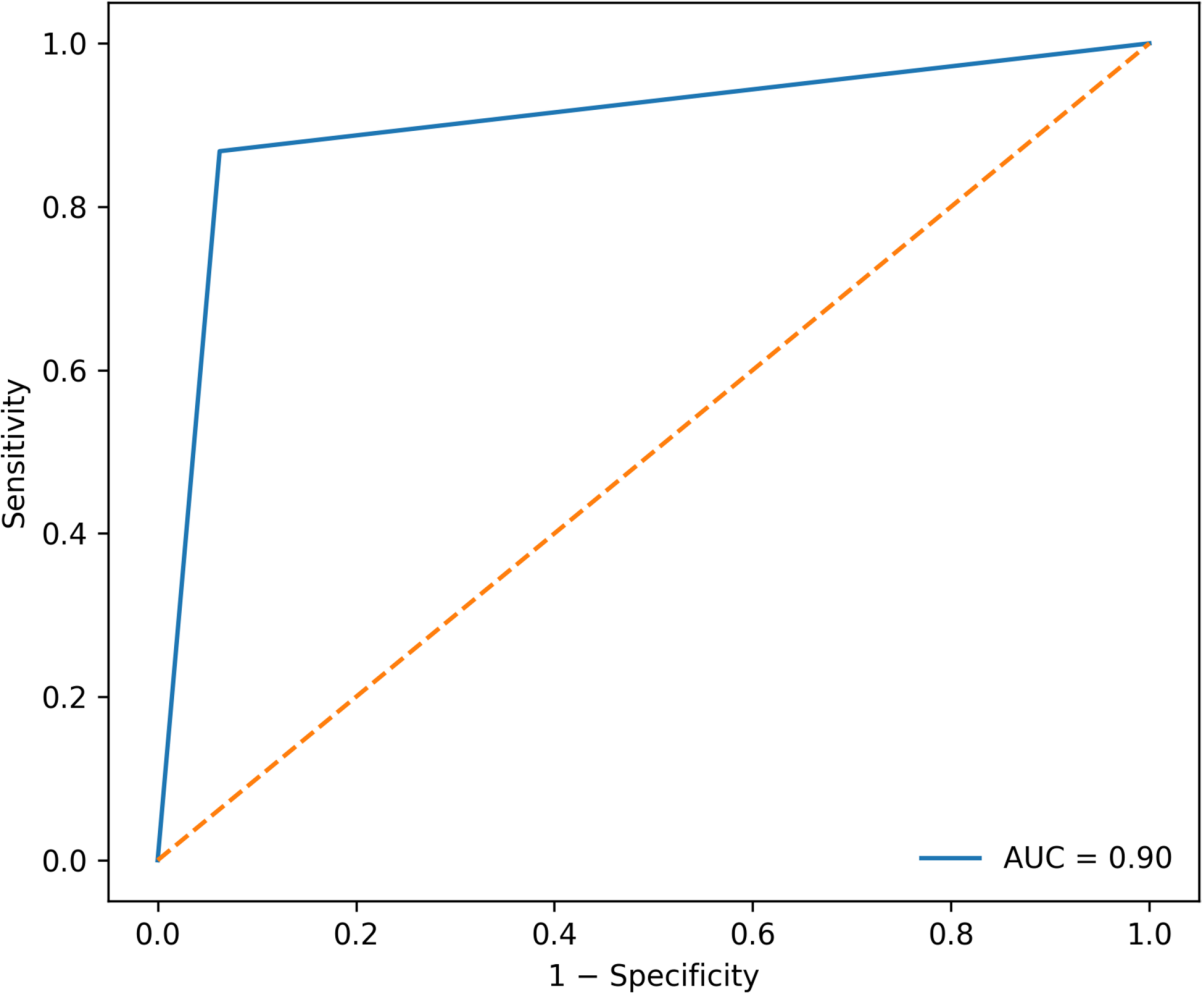
Receiver operating characteristic curve illustrating the performance of GPT in predicting the initial management strategy (tube thoracostomy versus conservative management) within the first 24 h. The area under the curve was 0.90

Receiver operating characteristic analysis was performed to evaluate the overall discriminative ability of GPT for predicting the initial management strategy within the first 24 h. The area under the receiver operating characteristic curve was 0.90 (95% CI, 0.84–0.96), indicating strong discrimination between patients managed with tube thoracostomy and those managed conservatively (Fig. 3).

## Discussion

Our study evaluated whether a vision-enabled generative pre-trained transformer (GPT) model could support clinically actionable interpretation of chest radiographs in spontaneous pneumothorax. We found that GPT aligned closely with clinician management decisions within the first 24 h and showed strong agreement with radiologist apical depth measurements. Importantly, the model’s misclassifications were not random: false positives often represented patients who later required intervention, while false negatives clustered around depth underestimation, suggesting a consistent and clinically interpretable error profile.

GPT showed clinically meaningful discrimination for initial management decisions. Contemporary pneumothorax care increasingly favors individualized, minimally invasive strategies in selected patients, but practice remains heterogeneous. The 2023 British Thoracic Society (BTS) pleural guideline emphasizes tailoring management to symptoms, physiologic stability, and imaging rather than relying on a single size threshold [3]. Large pragmatic trials also support less invasive pathways for primary spontaneous pneumothorax (PSP), including conservative management in appropriately selected patients and ambulatory approaches that reduce hospitalization at the expense of higher revisit or device-related events [8, 9]. Real-world surveys confirm that variation persists across emergency departments and specialty pathways, reflecting both clinical uncertainty and system-level constraints [1]. Against this backdrop, our results suggest that an image-only GPT output can approximate a key component of decision-making—radiographic severity—well enough to produce management recommendations that substantially match real clinical practice. We speculate that this performance arises because clinicians often use CXR severity (explicitly or implicitly) as an anchor, even when symptoms and comorbidities ultimately determine the final pathway.

GPT provided quantitative apical depth estimates that closely matched radiologist measurements, with a consistent tendency toward slight underestimation. Quantitative size estimation on chest radiography remains imperfect and may vary by projection, patient positioning, inspiration level, and reader experience. BTS guidance acknowledges these constraints and prioritizes pragmatic clinical integration over rigid size cutoffs [3]. Our findings add a new perspective: a multimodal generative model can deliver a reproducible numeric measurement that tracks radiologist assessment closely enough to support downstream clinical classification. The systematic underestimation we observed plausibly reflects the visual difficulty of identifying a thin pleural line at the apex in certain radiographs (e.g., low contrast, overlapping ribs/scapula, or marginal apical inclusion). Another plausible explanation is that the model weights global image cues (lung translucency gradients, mediastinal silhouette, and apical contour) differently than a human, which may bias it toward conservative numeric estimates in borderline cases. This directional bias matters clinically because underestimation preferentially increases false-negative management recommendations when depth approaches an intervention threshold. While the mean bias was modest, the Bland–Altman limits of agreement indicate that individual cases may show clinically meaningful underestimation of apical depth, which is most relevant near management thresholds. The model was not instructed or constrained to base its management recommendation solely on the numeric depth estimate and may also reflect global image features; nonetheless, depth underestimation represents an important potential failure mode. These findings support conservative guardrails and clinician review when outputs are used for decision support, especially in borderline cases.

The error pattern analysis provides clinically useful nuance beyond global performance metrics. False positives were uncommon and, notably, many of these patients ultimately underwent tube thoracostomy after 24 h. This finding suggests that GPT sometimes flagged patients who initially appeared suitable for observation but later demonstrated progression, symptoms, or persistent air leak that prompted intervention. Such “early warning” behavior could prove valuable in systems where follow-up, repeat imaging, or senior review is delayed. In contrast, false negatives occurred primarily when GPT underestimated apical depth relative to radiologists. We speculate that some false negatives also reflect the limits of an image-only approach: clinicians frequently intervene based on symptoms, oxygen requirement, comorbid lung disease, or concern for clinical deterioration—features that a radiograph alone cannot capture [1, 3]. From an implementation standpoint, these patterns argue for conservative guardrails if multimodal GPT outputs are used for decision support: for example, automatically escalating review when GPT reports low depth but other clinical risk signals are present, or when the image quality is suboptimal.

Most pneumothorax AI research has focused on detection and triage, often reporting strong diagnostic performance and potential workflow benefits [1]. Studies also show that AI assistance can improve clinician detection of pneumothorax on plain radiography, particularly among less experienced readers [4]. Parallel developments in generative AI suggest that models can draft radiographic reports with clinically comparable accuracy and may help flag urgent findings such as pneumothorax in real-world workflows [10]. Meanwhile, multimodal large language models trained to interpret chest radiographs, such as CXR-LLaVA, demonstrate the feasibility of report-like generation from images [7]. Our study extends this trajectory by evaluating a clinically “operational” task: generating both a numeric apical depth estimate and a management recommendation aligned to ED practice. This step from “is there a pneumothorax?” to “how large is it and what should we do now?” is precisely where bedside decision-making becomes complex—and where the safety requirements for AI support become stricter.

Clinical deployment should be limited to adjunct decision support within a human-in-the-loop workflow and not used for autonomous decisions. We recommend mandatory clinician review for all cases, conservative escalation when GPT recommends tube thoracostomy (or when estimated depth exceeds a prespecified alert threshold), and reassessment when outputs conflict with symptoms, oxygen requirement, or follow-up imaging. Outputs should be logged for auditability given potential model updates over time.

### Strengths and limitations

This study has several strengths. We evaluated a clear, clinically relevant endpoint (tube thoracostomy within 24 h) and paired it with a quantitative imaging output (apical depth), enabling both discrimination and agreement analyses. We also reported a structured error analysis, which provides practical insight into where and how the model fails—an important requirement for safe translation of AI tools into acute care workflows.

Several limitations warrant emphasis. First, this was a single-center retrospective study, which may limit generalizability to centers with different imaging protocols, patient mix, and practice thresholds. Second, we intentionally included only confirmed spontaneous pneumothorax cases because the study objective was to evaluate image-based depth estimation and management recommendation rather than pneumothorax detection. Nevertheless, this design likely inflates certain aspects of performance (e.g., laterality) and does not evaluate model behavior on normal radiographs or common mimics. Because ICC is influenced by between-subject variability, restricting the cohort to confirmed pneumothorax cases may overestimate agreement and reliability estimates compared with a diagnostically mixed cohort that includes normal or equivocal radiographs. This design likely inflates certain aspects of performance (e.g., laterality) and does not evaluate model behavior on normal radiographs or common mimics. Consequently, false-positive performance in real-world screening or triage settings remains untested, and this model should not be used for primary detection, screening, or triage in its current form. Third, we used clinical management within 24 h as the reference standard for the ‘need’ for tube thoracostomy. Although this reflects real-world decision-making, it does not represent a purely physiologic or imaging-defined severity threshold. Clinical management decisions may incorporate institutional practice patterns and patient-level considerations beyond radiographic findings. As a result, the reference standard represents a practice-informed clinical endpoint rather than an absolute measure of pneumothorax severity. Fourth, we did not provide symptom burden, oxygen requirement, comorbidity, or physiologic data to the model; therefore, our results reflect image-only decision support and cannot address whether multimodal integration with clinical variables would reduce false negatives. Fifth, measurement agreement relied on radiologist CXR-based depth estimation rather than CT-derived volumetric assessment. Because CT is typically performed in the supine position, whereas apical interpleural distance on upright PA radiographs reflects position-dependent air distribution, these metrics are not directly interchangeable. Although pragmatic and aligned with real-world emergency department practice, this approach may inherit interobserver variability and does not represent an absolute anatomic gold standard. Finally, vision-enabled GPT systems may change over time due to model updates, and prompt sensitivity can influence outputs—factors that mandate version control, auditability, and prospective evaluation before clinical deployment.

## Conclusion

In confirmed spontaneous pneumothorax, a vision-enabled GPT model produced apical depth estimates that agreed with radiologist measurements and management recommendations that aligned with initial ED decisions. These findings support its potential role as adjunct decision support, warranting prospective multicenter validation.

## Supplementary Information

Supplementary Material 1.

## Data Availability

The datasets generated and/or analyzed during the current study are not publicly available due to institutional and legal restrictions related to patient privacy and data governance. De-identified data may be made available by the corresponding author upon reasonable request and with the necessary institutional approvals.

## Abbreviations

LLM: Large language model
SP: Spontaneous pneumothorax
ED: Emergency department
CXR: Chest X-ray
