# Supplementary material for: Chest X-Ray evaluation using GPT for tube thoracostomy or conservative care in non-tension spontaneous pneumothorax
Source: Scand J Trauma Resusc Emerg Med. 2026 Apr 17;34:94. doi: 10.1186/s13049-026-01616-2 (PMC13214241; doi:10.1186/s13049-026-01616-2)
Supplement: Supplementary file 1 — Supplementary Material 1. [file 13049_2026_1616_MOESM1_ESM.docx]

You are evaluating a patient with confirmed spontaneous pneumothorax.

I will provide a posteroanterior (PA) chest radiograph along with the patient's age and sex.

No symptoms, vital signs, laboratory data, or outcome information will be provided.

Patient information:

- Age: [AGE]

- Sex: [SEX]

- Chest X-ray: (attached)

Based on the radiograph and demographic data, answer the following items in the exact format:

1. Laterality of pneumothorax (right/left)

2. Estimated apical pneumothorax depth in centimeters (Depth_cm)

3. Does this pneumothorax require tube thoracostomy, or is conservative management appropriate?

Answer ONLY in this three-line structured format:

Side: ___

Depth_cm: ___

Management: tube_thoracostomy / conservative
